# Sexual function in female business leaders: a cross-sectional study

**DOI:** 10.1590/1806-9282.20241293

**Published:** 2024-12-02

**Authors:** Renata Attili Maia, Gisele Vissoci Marquini, Manoel João Batista Castello Girão, Marair Gracio Ferreira Sartori, Zsuzsanna Ilona Katalin de Jarmy-Di Bella

**Affiliations:** 1 Universidade Federal de São Paulo, Department of Gynecology – São Paulo (SP), Brazil.

**Keywords:** Leadership, Professional competence, Motivation, Quality of life, Sexuality

## Abstract

**OBJECTIVE:**

The aim of the study was to provide a golden opportunity to access sexual function in women who act as leaders in companies through the use of an instrument validated for this purpose (the Female Sexual Function Index). The number of women in leadership positions has been gradually increasing, which shows the need for women to prepare themselves to reorganize the different aspects of life, including social, family, and personal factors as well as sexuality.

**METHODS:**

This is a cross-sectional study involving female executives employed in private companies who answered the Female Sexual Function Index. A convenience sample was made up of 50 women in leadership positions who had been employed for at least 1 year and were aged between 35 and 50 years in childbearing age.

**RESULTS:**

The mean total Female Sexual Function Index score of the evaluated sample was 22.6+7.6, characterizing sexual dysfunction, and the domain that was less negatively impacted was pain. It was also observed that all Female Sexual Function Index domains were worse in women between 41 and 50 years old (p<0.05), except desire.

**CONCLUSION:**

The total Female Sexual Function Index score of female company leaders was below the cutoff point for adequate sexual function. According to the results of this study and the applied methodology, professional success may not be associated with the quality of sexual life.

## INTRODUCTION

The female evolution is evident in the job market in recent decades, in the way of leading, in the globalized and dynamic world. The woman was naturally able to adapt to changes and perform well at several tasks at the same time. Thus, it was her merits that took her to new heights, merits that were equally evaluated in both men and women^1,2 3^.

Challenges caused by greater female participation go beyond issues related to leadership. Leadership, defined as interpersonal influence exercised in a situation directed through a human communication process with specific aims, is a typical phenomenon that occurs exclusively in social groups. The four elements that characterize leadership are the influence, the situation, the communication process, and the objectives to be achieved^2,3^.

Currently, some characteristics of female leaders, such as sensitivity, affectivity, versatility, and keen perception, among others, which until recently were considered weaknesses, have come to add up and are inspired by the productive process of organizations^3,4,5,6,7,8,9,10^. In this context, the process of feminization of the labor market may have enhanced sexual development. However, the active participation of women in leadership positions and their influence on female sexuality still deserves scientific clarification^3,4,5,6,7,8,9,10^.

Sexual health, defined by the World Health Organization as a physical, mental, and social state of well-being in relation to sexuality, adds the durability of affective relationships and the integral health and well-being of the individual. The lack of interest in sex, defined by the fifth edition of the Diagnostic and Statistical Manual of Mental Disorders (DSM-5) as sexual interest/arousal disorder, is one of the greatest challenges of female sexual dysfunction, probably also among business women^10,11,12,13,14,15^.

In this contemporary view, female leaders seek new expertise to meet the purposes and aspirations of companies/organizations, which seek excellence in results^8,9,16,17,18.19,20^. Female leaders still must deal with personal aspects, including the experience of sexuality with partnerships^21^. So far, there is a paucity of responses in the academic literature evaluating the sexuality of those women in leadership positions. Faced with this scenario, the objective of this study is to evaluate the sexual function of female business leaders.

## METHODS

### Participants and settings

This is a cross-sectional study involving executive women employed in private companies who answered the Female Sexual Function Index (FSF-I). The study was approved by the Ethics and Research Committee of Hospital São Paulo—UNIFESP, under number 5.825.858. A total of 120 women, occupying leadership positions (coordinators, supervisors, directors, managers, and chief executive officers), were invited through social networks online to answer the questionnaires. Inclusion criteria were age range from 35 to 50 years old, employed for at least 1 year, and sexually active (at least one relationship in the last 6 months) in a heterosexual relationship. Exclusion criteria were the presence of comorbidities (hypertension, diabetes, chronic diseases, and cancer undergoing treatment), menopause, and the use of antidepressant or anxiolytic medication. All women who were included signed the informed consent form before answering the FSF-I. Demographic data were collected through a questionnaire with direct questions and answers carried out by the main author.

The FSF-I is a written test that has six subscales and a sum of scores that measures the degree of each of the following domains: desire, excitement, lubrication, orgasm, satisfaction, and pain (dyspareunia), as a brief questionnaire that can be self-administered. A score greater than or equal to 26.55 is the cutoff to differentiate women without sexual dysfunction.

### Statistical analysis

Personal and work characteristics and quantitative sexuality scores were described using summary measures (mean, standard deviation, median, minimum, and maximum). Qualitative characteristics were described using absolute and relative frequencies^22^. The qualitative characteristics of the women were described according to age group using absolute and relative frequencies, and the existence of association with the group was verified using the chi-square test, while the sexuality scores and quantitative characteristics were described according to groups (women aged 35–40 and those over 40–50 years old) using summary and compared with the use of Mann-Whitney tests^22^.

The IBM-SPSS for Windows version 22.0 software was used to carry out the analyses, and Microsoft Excel 2013 software was used to tabulate the data. The tests were performed with a significance level of 5%.

### Definitions

“Heterosexual relationship”—only includes sexual preference according to sexual orientation without taking into account genetic sex or phenotype. “Steady partnership”—woman in a stable relationship where the partner participates in the daily tasks of taking care of the house or children without taking formal documentation into account^23^.

## RESULTS

Of the 120 women who received the invitation, 70 were not included because 32 declined the invitation by not responding, and the other 38 did not meet the established inclusion criteria. Thus, 50 sexually active women with leadership positions who completed the general questionnaire and the FSF-I were included. Table 1 shows the characteristics of the evaluated women, as well as the scores of the FSF-I domains and the total score. Then the sample was divided into two groups according to age: women aged 35–40 and those over 40–50 years old, and comparisons were made between these two groups in Table 2.

**Table 1:** Description of the characteristics of the evaluated women.

Variable	Description (n=50)
Age (years)	
Average±DP	40.9±4.6
Median (min; max)	40 (35; 50)
Age group, n (%)	
35–40	26 (52)
Over 40	24 (48)
Occupation, n (%)	
Administrator	14 (28)
Lawyer	11 (22)
Accountant	11 (22)
Economist	4 (8)
Psychologist	8 (16)
Publicist/advertising	2 (4)
Position held in company, n (%)	
CEO	2 (4)
Coordenator	13 (26)
Director	5 (10)
Manager	5 (10)
Supervisor	25 (50)
Time in the job position (years)	
Average±DP	3.64±2.44
Median (min; max)	3 (1; 11)
Sexual relations frequency per week, n (%)	
0	17 (34)
1	12 (24)
2	16 (32)
3	5 (10)
Have steady partner**, n (%)	
No	23 (46)
Yes	27 (54)
Number of children, n (%)	
0	7 (14)
1	11 (22)
2	26 (52)
3 or more	6 (12)
Desire	
Average±DP	3.3±1.18
Median (min; max)	3.6 (1.2; 6)
Excitement	
Average±DP	3.7±1.77
Median (min; max)	4.05 (0; 6)
Lubrification	
Average±DP	4.02±1.76
Median (min; max)	4.8 (0; 5.4)
Orgasm	
Average±DP	3.66±0.94
Median (min; max)	4 (2; 5.2)
Satisfaction	
Average±DP	3.67±1.82
Median (min; max)	3.8 (0.8; 6)
Pain	
Average±DP	4.2±1.82
Median (min; max)	5.4 (0.8; 6)
FSFI	
Average±DP	22.6±7.6
Median (min; max)	26.2 (6; 29)
Sexual dysfunction, n (%)	
No	22 (44)
Yes (FSFI≤26.5)	28 (56)

CEO: chief executive officer; DP: Desvio Padrão (standard deviation); FSFI: Female Sexual Function Index.

**Table 2:** Description of personal and work characteristics and sexuality scores according to age group.

Variable	Age group		p
	35–40	Over 40	
Time in the job position (years)			0.203
Average±DP	3.35±2.48	3.96±2.4	
Median (min; max)	2.5 (1; 10)	3.5 (1; 11)	
Sexual relations frequency per week, n (%)			**0.014**
0	5 (19.2)	12 (50)	
1	5 (19.2)	7 (29.2)	
2	14 (53.8)	2 (8.3)	
3	2 (7.7)	3 (12.5)	
Have steady partner, n (%)			0.266*
No	10 (38.5)	13 (54.2)	
Yes	16 (61.5)	11 (45.8)	
Number of children, n (%)			0.642
0	2 (7.7)	5 (20.8)	
1	6 (23.1)	5 (20.8)	
2	16 (61.5)	10 (41.7)	
3 or more	2 (7.7)	4 (16.7)	
Desire			
Average±DP	3.55±0.88	3.03±1.41	0.053
Median (min; max)	3.6 (1.2; 6)	2.4 (1.2; 5.4)	
Excitement			0.029
Average±DP	4.35±1.09	2.99±2.09	
Median (min; max)	4.8 (0.3; 6)	2.7 (0; 6)	
Lubrification			**<0.001**
Average±DP	4.79±1.06	3.19±2	
Median (min; max)	5.1 (0; 5.4)	4.2 (0; 5.1)	
Orgasm			**<0.001**
Average±DP	4.14±0.73	3.15±0.89	
Median (min; max)	4.4 (2; 5.2)	3.2 (2; 4.4)	
Satisfaction			**0.030**
Average±DP	4.25±1.37	3.05±2.07	
Median (min; max)	4.6 (0.8; 6)	2.4 (0.8; 6)	
Pain			0.039
Average±DP	4.83±1.48	3.52±1.93	
Median (min; max)	5.6 (0.8; 6)	2.6 (0.8; 6)	
FSFI			**0.001**
Average±DP	25.9±4.6	18.9±8.6	
Median (min; max)	27.6 (6.3; 29)	20.9 (6; 28.3)	

Mann-Whitney t-test; *chi-square test. Statistically significant values are denoted in bold. DP: Desvio Padrão (standard deviation); FSFI: Female Sexual Function Index.

As observed in Table 2, women over 40 years old had a lower frequency of sexual intercourse (p=0.014) in addition to worse scores in the different domains of the FSF-I (p<0.05). The desired domain (p=0.053) was almost statistically significant in line with the other domains. Thus, the total sexuality score (FSF-I) was lower in older women (p=0.001).

Table 3 shows that, separately, the older age group, not having a steady partner, and low frequency of weekly sexual intercourse were significantly related to the presence of sexual dysfunction according to the FSF-I (p<0.05). It was decided to adjust the final model with the age range and presence of a steady partner, and women over 40 years old had a 6.05 times greater chance of sexual dysfunction, as well as having a steady partner reduced the chance of sexual dysfunction measured by the FSF-I at 95% regardless of the other characteristics evaluated. Finally, as can be observed in Table 3, 38.5% of women between 35 and 40 years old presented sexual dysfunction by the FSF-I, and 75% of women between 40 and 50 years old.

**Table 3:** Description of the presence of sexual dysfunction according to personal and work characteristics and the result of unadjusted and adjusted analyses.

Variable	Sexual dysfunction		OR not adjusted	95%CI		p	OR adjusted	95%CI		p
	No	Yes		Inferior	Superior			Inferior	Superior	
Age group, n (%)						0.009*				**0.022**
35–40	16 (61.5)	10 (38.5)	1.00				1.00			
Over 40	6 (25)	18 (75)	4.8	1.42	16.19		6.05	1.29	28.28	
Time in the job position (years)			0.93	0.74	1.18	0.645				
Average±DP	3.86±2.51	3.46±2.41								
Median (min; max)	3 (1; 9)	3 (1.11)								
Sexual relations frequency per week, n (%)			0.42	0.22	0.79	**0.003**				
0	3 (17.6)	14 (82.4)								
1	4 (33.3)	8 (66.7)								
2	13 (81.3)	3 (18.8)								
3	2 (40)	3 (60)								
Have steady partner, n (%)						**<0.001**				**<0.001**
No	3 (13)	20 (87)	1.00				1.00			
Yes	19 (70.4)	8 (29.6)	0.06	0.02	0.27		0.05	0.01	0.27	
Number of children, n (%)			0.77	0.40	1.49	0.489				
0	2 (28.6)	5 (71.4)								
1	5 (45.5)	6 (54.5)								
2	12 (46.2)	14 (53.8)								
3 or more	3 (50)	3 (50)								

Mann-Whitney test; *chi-square test; logistic regression model for adjustment. Statistically significant values are denoted in bold. OR: odds ratio; CI: confidence interval; DP: Desvio Padrão (standard deviation).

## DISCUSSION

This is the first study that proposes to investigate the sexuality of female leaders in companies. The methodology analyzed women in the age group of 35–50 years old (more prevalent among executive women and often corresponds to the peak of their careers). Postmenopausal women were excluded from this study, as hormonal issues could interfere with sexuality.

The main finding of the study is that the sexual function score, using the FSF-I tool, in female leaders of private companies was considered below ideal (mean 22.6+SD 7.6), and the cutoff point for sexual dysfunction is a score equal to or less than 26.5. It was also observed that with increasing age, these values decreased even more, and in the age group from 41 to 50 years old, the mean was 18.9+8.6, with high rates of sexual dysfunction in women, even young and healthy women.

The results of the present study are in line with other studies on the subject. Sexual function was observed using two questionnaires, the FSF-I and the Functional Short Assessment Scale of Female Sexual Desire Dysfunction, and sexual dysfunction was found in 44.6% of women with a mean age of 32±3 years old, who were healthy and sexually active^24^.

Studies using the FSF-I have shown that women in the age range between maturity and old age demonstrated high levels of sexual dysfunction, with approximate scores of 60 to 67%. All FSF-I domains (desire, arousal, lubrication, orgasm, satisfaction, and pain) had low scores. The domains excitement, orgasm, and pain were the ones that most contributed to the low FSF-I scores^25,26^.

On the contrary, it is possible that sexual dysfunction becomes more frequent in the postmenopausal period due to organic and functional issues triggered by hypoestrogenism, which was not the scope of this study. Using the MRS scale (Menopause Rating Scale), studies observed that climacteric symptoms, including hot flashes, depressive mood, and vaginal dryness, seem to influence the sexual function of women^6,25,26^. Although it is difficult to measure sexuality and even sexual dysfunction, many parameters deserve to be considered, not only age or climacteric period. Some factors can vary greatly in the same individual, and even more so, considering the sexual partner^24^. Sexuality is a set of special characteristics, external or internal, favorable for maintaining health in general, both in physical and mental aspects, also in menopause^26^.

In addition, sexuality contains within itself the experience of meaning, being a human dimension, dynamic and dialectical, procedural, mutable, and historical, always open to new forms of meaning^12,13^. The concept, which includes aspects of genitalia, is not limited to it; in other words, it concerns feelings, emotions, pleasures, and libidinal eroticism involved in interpersonal relationships, which may or may not include sexual relationships between individuals^13,14^.

Relationships refer to leadership, which is an interpersonal influence in which one acts to intentionally modify the behavior of another^4^. Generally, influence involves concepts such as power and authority, encompassing all the ways in which they introduce changes in the behavior of people or groups of people^3,4,5^. In some studies, it is stated that leadership is everyone’s business, and it does not depend on power or formal authority. Leadership involves relationships, credibility, and actions^7^.

Although it is expected, in a few years, the predominance of women in the leadership of public and private organizations, there can be considerable variations in this percentage according to the socio-economic levels of the countries. Studies carried out in the area of economics and the business sector demonstrate that women make up 41% of the workforce but occupy only 24% of management positions^9,10^. On the contrary, a recent study observed that the share of women in executive positions at the 300 largest sample of worldwide companies rose from 8% in 1990 to 13% in 2000. In terms of income, generally, women can receive, on average, the equivalent of 71% of men’s salaries. This difference is more evident in less qualified positions. At the top, they almost reach the men. Studies show that in the world of work, women are still preferred for routine functions^9,10,11^.

In this context, a leader is a person who guarantees that the team members are led by someone who is highly inspiring and motivating, and as a natural consequence, considering the culture and strategic planning of each organization^8^. However, this interpersonal relationship does not always seem to be the same in sexuality.

As a parameter of sexuality, libido is the term used in the theory of drives to demonstrate the dynamic manifestation of sexuality^20^. Libido is the energy that underlies the transformations of the sexual drive; it has been found that women who hold leadership positions in companies, although successful, have low sexual satisfaction and/or sexual dysfunction^19^. In a population study, for a large proportion of women, including younger ones, nothing is lower than their libido, with a third of those interviewed finding it difficult to be interested in sex^19^.

Therefore, sexuality is continually influenced by the social processes that organize the structure and expression of desire^12^. Sexual satisfaction is essential for well-being. Numerous studies have demonstrated the relationship between satisfactory sex life and greater capacity to work and to love, satisfaction and adaptation in intimate relationships, self-esteem, physical and mental health, quality of life, emotional satisfaction, happiness, and satisfaction with life, including career^9,16^. The impact that sexual satisfaction has on all these variables related to well-being and professional development justifies research that aims to establish its determinants, such as communication with the partner, attachment styles and/or insecurity, sexual frequency, and career success^9,11,17,19^.

### Strengths and limitations

One of the strengths of this article is its pioneering approach to studying the first aspects of sexuality in this specific group of business women. In addition, the application of a validated questionnaire with ethical approval for its realization demonstrates methodological rigor as a reproducibility instrument. The main limitation was the lack of robustness of the sample, precisely due to the logistics of optimizing business women’s time to dedicate themselves to answering scientific research.

## CONCLUSION

The total FSF-I score of female leaders in companies was below the cut-off point for adequate sexual function, especially in the age group over 40 years old. According to the results of this study and the applied methodology, professional success may not be associated with quality of sexual life. Future prospective and multicenter studies may clarify this association even more rigorously.
